# Type 1 diabetes mellitus induced by sintilimab: a case report and related case literature analysis

**DOI:** 10.3389/fendo.2026.1881986

**Published:** 2026-07-01

**Authors:** Hao Su, Xinyu Zhao, Huayu Liu, Mianli Li, Haitao Geng

**Affiliations:** Department of Oncology, Binzhou Medical University Hospital, Binzhou, Shandong, China

**Keywords:** cancer, diabetes ketoacidosis, immune-related adverse events, immunocheckpoint inhibitor related diabetes, sintilimab

## Abstract

Immune checkpoint inhibitors are widely used in the treatment of malignant tumors. Although the incidence rate of type 1 diabetes induced by these inhibitors is low, the damage to pancreatic β cells is irreversible and the treatment effect of steroids is poor, which requires clinical attention. This article reports a case of a 69-year-old patient with poorly differentiated adenocarcinoma of the stomach and colon who developed this disease during treatment with Sintilimab combined with the SOX regimen. After insulin treatment, her blood glucose levels gradually stabilized. Based on the literature, we explore the pathogenesis, diagnostic difficulties, and current treatment status centered around insulin replacement therapy. We emphasize the need for clinicians to strengthen blood glucose monitoring during treatment with immune checkpoint inhibitors, identify symptoms early, and optimize diagnosis and treatment to improve patient prognosis.

## 1 Introduction

In the evolution of tumor treatment methods, the emergence of immune checkpoint inhibitors (ICIs)—including programmed cell death protein 1 (PD-1)/programmed death ligand 1 (PD-L1) inhibitors and cytotoxic T lymphocyte antigen 4 (CTLA-4)—has provided a new approach for the treatment of malignant tumors, and at the same time, it signifies a deeper understanding of the human immune system and cancer treatment. Since the launch of the first ICIs drug for the treatment of melanoma in 2011, an increasing number of ICIs have been used in more and more clinical trials. However, as clinical trials progress, immune-related adverse events (irAEs) have also emerged. IrAEs typically refer to the immune or inflammatory response caused by the activation of the immune system, which can affect almost all organs and systems, including pneumonia, colitis, hepatitis, dermatitis, nephritis, pancreatitis, vitiligo, pruritus, and endocrine diseases, such as thyroiditis, hypophysitis, primary adrenal insufficiency, and type 1 diabetes (T1DM) (1).

Among them, chronic irAEs involving the endocrine system are the earliest reported type, with an incidence rate of 15%-40% in patients treated with ICIs. However, these irAEs are irreversible and insensitive to steroid treatment, especially in ICIs-induced type 1 diabetes mellitus (ICI-T1DM). Despite the low incidence rate of this disease (0.2%-1.4%), steroid treatment not only fails to reverse the loss of β-cell function in the pancreatic islets but also exacerbates insulin resistance (2–5). This paper aims to analyze the clinical diagnosis and treatment process of a patient with ICI-T1DM induced by Sintilimab, combined with the literature data of ICI related diabetes, summarize the clinical characteristics, pathogenesis and management strategies of ICI related diabetes, provide reference for clinical prevention and treatment of ICI related autoimmune diabetes, and promote the clinical rational drug use of tumor immunotherapy.

## 2 Case report

**Figure 1:**
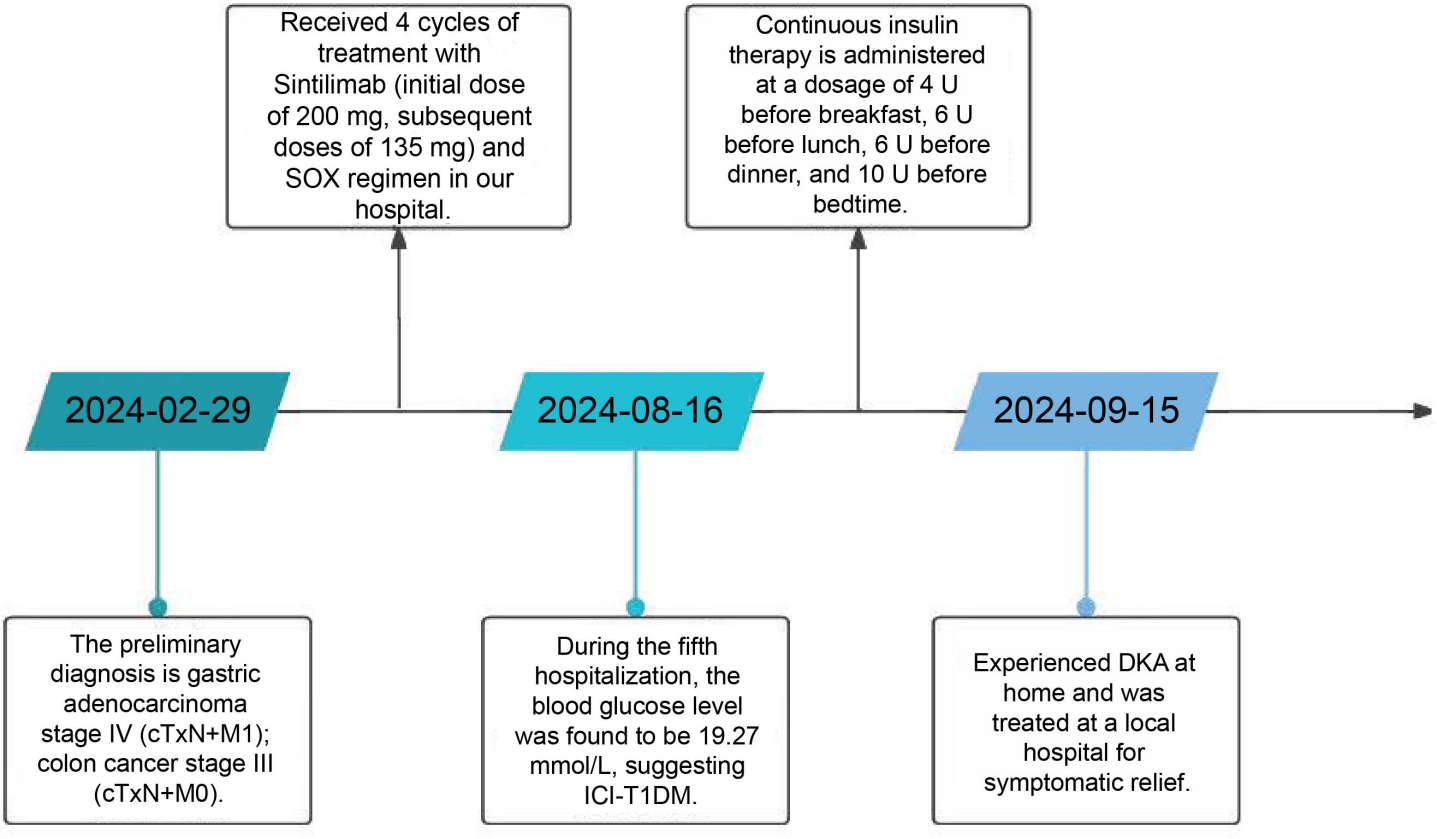
Key points of patient’s disease progression.

A 69-year-old female patient had no basic medical history and family history of hypertension, diabetes, heart disease, *etc.*, no history of surgery, food and drug allergy, and her BMI was 23.31 kg/m^2^ when she was initially diagnosed as poorly differentiated adenocarcinoma of stomach and colon. On February 29, 2024, she was diagnosed with poorly differentiated adenocarcinoma of the stomach and colon and received 4 cycles of treatment with Sintilimab (initial dose of 200 mg, subsequent doses of 135 mg) and SOX regimen (Oxaliplatin and Tegafur, Gimeracil and Oteracil Potassium Capsules) in our hospital. During the fifth admission for the next cycle of immunotherapy on August 16, 2024, laboratory tests revealed a blood glucose level of 19.27 mmol/L, glycosylated hemoglobin of 7.5%, Serum β-hydroxybutyrate of 2.59 mmol/L, and adrenocorticotropic hormone of 14.480 pmol/L. Two diabetes-related autoantibodies, including anti-glutamic acid decarboxylase antibody and anti-insulin antibody, showed no remarkable abnormalities. Given that the patient had no history of diabetes, ICI-T1DM was immediately considered. Immediately perform fluid replacement and subcutaneous injection of 6 U human insulin injection. After the blood sugar stabilizes, the treatment is changed to 8 U and 11 U of insulin glargine for breakfast and bedtime respectively, and 4 U of human insulin injection for lunch and dinner. Additionally, one dose of SOX regimen was administered. At discharge, blood glucose was well controlled (fasting 4.0 mmol/L, 11.3 mmol/L after breakfast, 9.0 mmol/L after lunch). After being re-admitted for one round of SOX chemotherapy, the patient presented to a local hospital due to diabetic ketoacidosis during the outpatient period. During the final hospitalization period of the SOX regimen, the insulin lowering regimen was adjusted to use 4 U and 10 U of glargine insulin for breakfast and bedtime, respectively, and 6 U of human insulin injection before lunch and dinner. On September 15, 2024, the patient experienced diabetic ketoacidosis (DKA) at home and was treated at a local hospital for symptomatic relief. The patient received the first dose of sintilimab on March 6, 2024. Hyperglycemia, indicating the onset of drug-induced diabetes mellitus (DM), was first detected on August 16, 2024. The time interval from initial sintilimab administration to DM onset was approximately 163 days (5.4 months). Up to now, the patient has been maintained on insulin therapy, and insulin has not been discontinued (**Figure 1**). The main blood test indexes of the patient can be seen in **Table 1**.

**Table 1:** Main blood test indexes.

Inspection items	Time	Value	Reference range
Blood glucose, mmol/L	2024-02-29	6.42	3.9-6.1
	2024-04-26	5.25	
	2024-05-15	5.99	
	2024-08-16	19.27	
	2024-08-20	4.00	
	2024-12-26	5.68	
C-peptide, ng/ml	2024-08-20	0.12	1.1-4.4
Glycosylated hemoglobin, %	2024-08-20	7.50	4-6
Serum β-hydroxybutyrate, mmol/L	2024-08-20	2.59	0.03-0.3
Anti-GAD antibodies, IU/ml	2024-08-20	0.89	0-10
	2024-12-26	1.10	
Anti-insulin antibody, COI	2024-08-20	0.09	0-1.1
	2024-12-26	0.35	

## 3 Discussion

This 69-year-old female patient had no prior history of diabetes. Severe hyperglycemia with elevated serum β-hydroxybutyrate emerged during sintilimab treatment, suggestive of acute pancreatic β-cell injury secondary to PD-1 inhibitor therapy. Combined with the clear temporal relationship between drug exposure and acute glycemic deterioration, the patient was diagnosed with ICI-T1DM. A prominent serological distinction exists between ICI-T1DM and classic autoimmune type 1 diabetes: classic type 1 diabetes shows an islet autoantibody positive rate of 60%-90%, while ICI-T1DM typically presents negative anti-GAD and anti-insulin antibodies, consistent with the seronegative profile observed in this case. A normal adrenocorticotropic hormone level further excluded concurrent secondary adrenal insufficiency. For differential diagnosis, acute exacerbation of occult type 2 diabetes was ruled out given the absence of chronic metabolic disorders. Classic fulminant type 1 diabetes was also excluded, as its typical autoantibody positivity and predisposing factors were absent in this patient. The close temporal correlation between sintilimab administration and acute hyperglycemia confirmed the final diagnosis of ICI-T1DM.

T1DM is an autoimmune disease jointly caused by genetics and environment, specifically manifested as the destruction or dysfunction of pancreatic β-cells due to abnormal activation of the immune system (6). In the pathogenesis of this disease, self-activated T lymphocytes play a pivotal role (7). Firstly, macrophages and dendritic cells infiltrate the pancreatic islets and migrate to the pancreatic lymph nodes, where they present antigens from pancreatic β-cells and activate CD4+ T lymphocytes. The activated CD4+ T lymphocytes, together with macrophages and dendritic cells, promote the migration of CD8+ T lymphocytes to the pancreatic islets. Ultimately, the combined action of CD4+ and CD8+ T lymphocytes leads to the death of pancreatic β-cells (8, 9).

PD-1 is expressed on the surface of T cells, and when it binds to PD-L1, it transmits inhibitory signals to maintain immune tolerance. However, PD-1 is not only expressed on the surface of lymphocytes; it also exists in target organs such as pancreatic β-cells. The use of ICIs blocks the interaction between PD-1 and PD-L1, which leads to the activation and proliferation of autoreactive T cells. These autoreactive T cells can activate macrophages by releasing interferons (IFNs) and kill pancreatic β-cells using nitric oxide, ultimately resulting in insulin deficiency and ICI-T1DM in patients (10, 11). Multiple animal experiments have confirmed the regulatory effect of the PD-1/PD-L1 pathway on ICI-T1DM. Treatment with PD-1 inhibitors can rapidly induce the onset of ICI-T1DM in adult non-obese diabetic (NOD) mice (12). Mice with PD-1 or PD-L1 gene knockout exhibit a markedly earlier onset of ICI-T1DM (13). Meanwhile, blockade of the PD-1/PD-L1 pathway directly triggers ICI-T1DM in the antigen-specific immune tolerance model (14). Additional mechanistic studies further verify the pivotal role of the PD-1/PD-L1 pathway in the progression of ICI-T1DM. Interferon-gamma (IFN-γ) can upregulate PD-L1 expression in pancreatic β-cells of NOD mice (15). Loss of PD-1 in CD4^+^ T cells dramatically enhances islet antigen-specific immune infiltration and exacerbates immune damage to pancreatic islets. In conclusion, dysregulation of the PD-1/PD-L1 signaling pathway serves as a critical mechanism underlying the initiation and progression of ICI-T1DM.

Currently, the diagnosis of this disease remains challenging. Non-diabetics lack data on glycosylated hemoglobin testing, making it difficult to distinguish between worsening type 2 diabetes and newly diagnosed ICI-T1DM. Moreover, autoantibody testing for ICI-T1DM cannot serve as a definitive diagnostic tool, and early detection may even interfere with the diagnosis of the disease. Notably, immune checkpoint inhibitors may trigger multiple endocrine irAEs simultaneously. Therefore, we further detected adrenocorticotropic hormone levels during hyperglycemia to screen for concurrent secondary adrenal insufficiency. For example, in the patient we are discussing, when blood glucose levels were first elevated, there was no significant increase in anti-glutamic acid decarboxylase antibody, anti-insulin antibody, or adrenocorticotropic hormone levels. Furthermore, the time of onset varied greatly. In different clinical studies, the median time to diagnosis of ICI-T1DM ranged from 7 to 25 weeks, and 40%-76% of patients experienced DKA as their initial onset (16–18). According to research data, significant destruction of pancreatic β-cells and the onset of hyperglycemic symptoms can occur as early as 5 days after the initial medication administration, and as late as several months after drug discontinuation (19, 20). Currently, authoritative institutions in the fields of endocrinology and oncology have called on medical practitioners to pay attention to ICI-T1DM, recommending fasting or random blood glucose testing and HbA1c testing before and after each administration of anti-PD-1 therapy.

Moreover, developing specific diagnostic testing methods is what we urgently need at present (21, 22). For example, in patients with T1DM, by monitoring the demethylation of insulin genes (INS1 and INS2) in peripheral blood, it is possible to detect the destruction of pancreatic β-cells before blood glucose levels become uncontrolled. However, whether this method is applicable to ICI-T1DM still requires further verification (23, 24). From the perspective of clinical feasibility, the INS1/INS2 demethylation assay has both research value and inherent defects. Its advantage lies in the ability to capture early acute β-cell death prior to obvious glycemic abnormalities. However, the transient nature of the target signal sets a strict requirement for sampling timing, limiting its application in random clinical testing. Meanwhile, the complicated experimental procedures and expensive testing costs also hinder its widespread use in daily clinical work. Currently, this technique can be applied as an auxiliary indicator for clinical research and high-risk population screening. Nevertheless, immediate and accessible blood glucose detection is still irreplaceable for routine monitoring and real-time safety warning of patients. Although human leukocyte antigen (HLA)-related susceptibility genes are associated with ICI-T1DM, their value as precise screening indicators is limited. The incidence of ICI-T1DM is less than 1%, and the low baseline prevalence limits the positive predictive value; Existing research has not systematically reported key screening indicators such as sensitivity and specificity. There are racial differences in HLA frequency among populations, and multiple pathogenic mechanisms limit the role of a single biomarker. Therefore, HLA testing should be used as an auxiliary reference tool and cannot be used alone for precise screening and diagnosis (25).

The current management strategies for ICI-T1DM are primarily established through experience gained from clinical practice. Clinically, insulin is commonly used as the standard treatment for hyperglycemia control, accompanied by some auxiliary supportive treatments such as fluid replacement and correction of electrolyte imbalance (26, 27). So far, no effective preventive measures have been found to avoid the occurrence of ICI-T1DM. Furthermore, clinical studies by Aleksova et al. suggest that even with high-dose corticosteroids for immune modulation intervention, the progression of autoimmune diabetes cannot be reversed (28). Although the measurement of C-peptide is not a necessary item for the diagnosis of ICI-T1DM, the values of C-peptide obtained through continuous measurement in clinical practice may provide assistance in treatment (29). At the same time, for patients receiving ICIs treatment, Al-Taie et al. (30) suggested that clinicians should carry out patient education, close monitoring, appropriate screening and follow-up before immunotherapy, which would help to identify patients with hyperglycemia or new onset diabetes due to treatment, so as to improve the health status and quality of life of patients. In addition, the guidelines suggest that patients with grade 3 hyperglycemia or T1DM should suspend the use of Sintilimab before they need to receive Sintilimab treatment, while patients with grade 4 hyperglycemia need to permanently stop treatment, continue blood glucose monitoring and implement appropriate insulin replacement therapy. Overall, the treatment pathway for ICI-T1DM is similar to that of T1DM, with patients generally relying on insulin injections to reduce blood glucose levels. The patient we are discussing has been dependent on insulin for glucose-lowering therapy since being diagnosed with ICI-T1DM. However, considering that such patients often have advanced malignant tumors and a poor overall prognosis, individualized glycemic management strategies should prioritize the prevention of diabetic ketoacidosis. Intensive basal-bolus regimens or continuous insulin infusion pumps are recommended for treatment, and overly strict glycemic targets should be avoided to lower the risk of severe hypoglycemia. Although there is currently a lack of specific guidance documents for ICI-T1DM, based on a comprehensive assessment of risks and benefits, clinical practice recommends referring to the management framework for “T1DM patients with comorbid malignancies”. In this context, insulin regimens still occupy a central position in the overall treatment management of patients.

Accumulated case reports and literature reviews have confirmed diabetes mellitus as a typical immune-related adverse reaction associated with sintilimab therapy. Zan et al. (31) reported a patient with gastric adenocarcinoma who developed sintilimab-induced DM complicated with thyroid dysfunction after receiving this PD-1 inhibitor. Similarly, Huang et al. (32) documented another case of sintilimab-related DM accompanied by psoriasis, further verifying the causal relationship between sintilimab exposure and new-onset DM. These published cases and retrospective literature analyses provide solid evidence to support our clinical observation. Collectively, these studies indicate that sintilimab has the potential to induce heterogeneous endocrine and cutaneous irAEs, among which new-onset diabetes mellitus deserves close clinical monitoring during medication.

## 4 Conclusion

Despite the low incidence of ICI-T1DM, the widespread use of various ICIs has rendered this irAEs a non-negligible clinical concern. As demonstrated in this sintilimab-associated ICI-T1DM case, the disease manifests months after treatment commencement, which suggests that the pathogenesis is multifactorial rather than solely attributed to PD-1/PD-L1 blockade, and the relevant co-factors have not been fully clarified to date. Currently, effective biomarkers to identify susceptible populations are absent. HLA-related susceptibility cannot serve as an accurate screening indicator. Under the current circumstances with no established prevention protocols, we put forward persistent clinical recommendations: implement regular blood glucose monitoring for all patients on ICI therapy, improve vigilance for hyperglycemia, and prioritize DKA prophylaxis. These strategies are vital to reduce adverse clinical outcomes of ICI-T1DM.

## Data Availability

The original contributions presented in the study are included in the article/supplementary material. Further inquiries can be directed to the corresponding author.
